# Treatment outcomes and prognostic factors in patients with driver mutant non-small cell lung cancer and de novo brain metastases

**DOI:** 10.1038/s41598-024-56046-w

**Published:** 2024-03-09

**Authors:** Seda Kahraman, Serdar Karakaya, Muhammed Ali Kaplan, Sema Sezgin Goksu, Akin Ozturk, Zehra Sucuoglu Isleyen, Jamshid Hamdard, Sedat Yildirim, Tolga Dogan, Selver Isik, Abdussamet Celebi, Burcu Belen Gulbagci, Nail Paksoy, Mutlu Dogan, Haci Mehmet Turk, Ahmet Bilici, Ali Murat Tatli, Sinem Akbas, Nedim Turan, Ilhan Hacibekiroglu, Gamze Gokoz Dogu, Adnan Aydiner, Ahmet Taner Sumbul, Serap Akyurek, Merih Yalciner, Ahmet Demirkazik, Pinar Gursoy, Musa Baris Aykan, Elif Sahin, İbrahim Karadag, Osman Kostek, Muhammed Muhiddin Er, Mehmet Artaç, Yakup Duzkopru, Dincer Aydin, Deniz Isik, Yusuf Karakas, Saadettin Kilickap, Cihan Erol, Bilgin Demir, Burak Civelek, Yakup Ergun, Muhammed Bulent Akinci, Izzet Dogan, Nuri Karadurmus, Perran Fulden Yumuk, Mehmet Ali Nahit Sendur

**Affiliations:** 1 Department of Medical Oncology, Ankara Bilkent City Hospital, Ankara Yildirim Beyazit University, 06800 Ankara, Turkey; 2 Department of Medical Oncology, Ankara Ataturk Sanatoryum Training and Research Hospital, Ankara, Turkey; 3 Department of Medical Oncology, Dicle University Medical Faculty Hospital, Diyarbakir, Turkey; 4 Department of Medical Oncology, Akdeniz University Hospital, Antalya, Turkey; 5 Department of Medical Oncology, Sureyyapasa Chest Diseases and Chest Surgery Training and Research Hospital, Istanbul, Turkey; 6 Department of Medical Oncology, Faculty of Medicine Hospital, Bezmialem Vakif University, Istanbul, Turkey; 7 Department of Medical Oncology, Medipol University Hospital, Istanbul, Turkey; 8 Department of Medical Oncology, Kartal Dr. Lutfi Kirdar City Hospital, Istanbul, Turkey; 9 Department of Medical Oncology, Pamukkale University Medical Faculty Hospital, Denizli, Turkey; 10 Department of Medical Oncology, Marmara University Pendik Training and Research Hospital, Istanbul, Turkey; 11 Department of Medical Oncology, Sakarya University School of Medicine, Sakarya, Turkey; 12 Department of Medical Oncology, Istanbul Faculty of Medicine, Istanbul University, Istanbul, Turkey; 13 Department of Medical Oncology, Ankara Dr Abdurrahman Yurtaslan Oncology Training and Research Hospital, Ankara, Turkey; 14 Department of Medical Oncology, Koç University Medical Faculty Hospital, Istanbul, Turkey; 15 Department of Medical Oncology, Baskent University Adana Dr. Turgut Noyan Application and Research Center, Adana, Turkey; 16 Department of Radiation Oncology, Faculty of Medicine, Ankara University, Ankara, Turkey; 17 Department of Medical Oncology, Faculty of Medicine, Ankara University, Ankara, Turkey; 18 Department of Medical Oncology, Ege University Medical Faculty Hospital, Izmir, Turkey; 19 Department of Medical Oncology, Gulhane Training and Research Hospital, Ankara, Turkey; 20 Department of Medical Oncology, Kocaeli University Medical Faculty Hospital, Kocaeli, Turkey; 21 Department of Medical Oncology, Hittite University Corum Training and Research Hospital, Corum, Turkey; 22 Department Of Medical Oncology, Necmettin Erbakan University Meram Medical Faculty Hospital, Konya, Turkey; 23 Department of Medical Oncology, Ankara Etlik City Hospital, Ankara, Turkey; 24 Department of Medical Oncology, Kocaeli Derince Training and Research Hospital, Kocaeli, Turkey; 25 Department of Medical Oncology, Kocaeli Medical Park Hospital, Kocaeli, Turkey; 26 Department of Medical Oncology, Acıbadem Bodrum Hospital, Mugla, Turkey; 27 Department of Medical Oncology, Liv Hospital, Istinye University, Ankara, Turkey; 28 Department of Medical Oncology, Aydin Ataturk State Hospital, Aydin, Turkey; 29 Department of Medical Oncology, Ankara Bilkent City Hospital, Ankara, Turkey; 30 Department of Medical Oncology, Batman Training and Research Hospital, Batman, Turkey; 31 Department of Medical Oncology, Basaksehir Cam and Sakura City Hospital, Istanbul, Turkey

**Keywords:** Oncogene-driven advanced non-small cell lung cancer, De novo brain metastases, Survival related parameters, Cancer, Oncology

## Abstract

Central nervous system (CNS) metastases can be seen at a rate of 30% in advanced stages for patients with non-small cell lung cancer (NSCLC). Growing evidence indicates the predictive roles of driver gene mutations in the development of brain metastases (BM) in recent years, meaning that oncogene-driven NSCLC have a high incidence of BM at diagnosis. Today, 3rd generation targeted drugs with high intracranial efficacy, which can cross the blood–brain barrier, have made a positive contribution to survival for these patients with an increased propensity to BM. It is important to update the clinical and pathological factors reflected in the survival with real-life data. A multi-center, retrospective database of 306 patients diagnosed with driver mutant NSCLC and initially presented with BM between between November 2008 and September 2022 were analyzed. The median progression-free survival (mPFS) was 12.25 months (95% CI, 10–14.5). While 254 of the patients received tyrosine kinase inhibitor (TKI), 51 patients received chemotherapy as first line treatment. The median intracranial PFS (iPFS) was 18.5 months (95% CI, 14.8–22.2). The median overall survival (OS) was 29 months (95% CI, 25.2–33.0). It was found that having 3 or less BM and absence of extracranial metastases were significantly associated with better mOS and iPFS. The relationship between the size of BM and survival was found to be non-significant. Among patients with advanced NSCLC with de novo BM carrying a driver mutation, long-term progression-free and overall survival can be achieved with the advent of targeted agents with high CNS efficacy with more conservative and localized radiotherapy modalities.

## Introduction

Central nervous system (CNS) metastases can be seen at a rate of 30% in advanced stages for patients with non-small cell lung cancer (NSCLC). While 10–25% of patients with stage 4 NSCLC present with BM at diagnosis, the first site of recurrence has been shown to be BM in approximately 20% of patients after definitive treatment for unresectable stage 3 disease^1–3^. CNS metastasis is one of the main causes of death and contributes to dismal prognosis as an unfortunate site of disease progression. Also, BM is closely associated with poor performance status, quality of life and morbidity in these patients.

Nevertheless, growing evidence indicates the predictive roles of driver gene mutations in the development of BM in recent years, meaning that oncogene-driven NSCLC have a high incidence of BM at diagnosis^4–10^. A recent retrospective study reported that patients with NSCLC with diverse targetable molecular alterations, except for the KRAS mutation which was excluded in the study and who had received targeted systemic therapies, had a high incidence of BM at diagnosis^11^. Interestingly, they also found that patients with BM at the time of diagnosis and who subsequently developed BM and receiving local and systemic treatment had similar outcomes when compared to the patients without BM. Today, 3rd generation targeted drugs with high intracranial efficacy, which can cross the blood–brain barrier, have made a positive contribution to survival for these patients with an increased propensity to BM.

Specifically, regarding BM in patients with EGFR mutation or ALK rearrangement, 3rd generation EGFR TKI osimertinib and 2nd and 3rd generation ALK TKIs revealed significant risk reductions for CNS disease progression when compared to their first-generation counterparts^12–16^.

Stereotactic radiosurgery (SRS), whole-brain irradiation (WBRT) and surgical resection are applied alone or combined/sequentially as local treatment strategies of CNS metastases for the patients with driver mutant NSCLC. In this patient group, it is crucial to investigate factors such as the type of driver mutation, the number of BM, clinicopathological features of the patients, the sequence of the treatment regimens they receive, and the relationship of these factors with life expectancy of the patients and adherence to treatment.

Due to limited data in the literature, we aimed to evaluate the outcome of the patients diagnosed with driver mutant NSCLC who had de novo BM as stage 4 disease and a number of key features including TKI treatments and systemic treatments, patient characteristics, treatments for CNS control, as well as prognostic factors related to intracranial progression-free and overall survival time of these patients.

## Materials and methods

The study was initiated as a Turkish Oncology Group (TOG) project. A multi-center (31 tertiary care oncology centers), institutional review board-approved, retrospective database of 306 patients diagnosed with driver mutant NSCLC and initially presented with BM between between November 2008 and September 2022 were analyzed. The patients included were required to receive at least one line of targeted therapy. The patients who subsequently developed BM were excluded. Clinicopathologic characteristics including age at diagnosis, gender, smoking status, Eastern Cooperative Oncology Group (ECOG) performance status (PS), tumor histology, present targetable driver mutations, number and the size of the largest one of intracranial metastasis, extracranial metastatic status, local treatments for BMs and systemic treatments, treatment discontinuation, presence and time of progression and death were noted.

Adverse events obtained from medical records were graded using the National Cancer Institute Common Terminology Criteria for Adverse Events (CTCAE) version 4.03. The study protocol was approved by the ethics committee of Ankara Bilkent City Hospital as a multicenter retrospective observational study and all methods were carried out in accordance with Declaration of Helsinki.

Since no experimental procedures were performed on patients and data were retrospectively collected from the medical files of the patients; the need for informed consent was waived by our institutional review board, that is Ankara Bilkent City Hospital, Ethics Committee Number 2, which is deemed unnecessary according to national legislation.

### Statistical analysis

The progression-free survival (PFS) was defined as the duration of time from initial first-line treatment to disease progression or the most recent follow-up. And the intracranial PFS (iPFS) was measured from the date of initial first-line treatment to the date of BM progression. The overall survival (OS) was calculated as the time interval in months between de novo BM diagnosis and death or loss of follow-up, whichever was earlier.

Kaplan–Meier method was used to analyse survival data and the log rank test were performed to compare the differences. Multivariable Cox regression analysis was used to determine potential prognostic factors for OS and iPFS. Statistical significance level was determined at P < 0.05. SPSS Statistics version 26.0 was used for the analysis.

## Results

Analysis included 306 patients diagnosed with driver mutant, de novo brain metastatic NSCLC. The median age of the patients was 58 (20–85) years. There were 155 female (50.7%), 151 male (49.3%) patients. Most of the patients were never-smoker (58%) and had ECOG PS of 0–2 (94.3%). Regarding tumor-related features, 94.4% of them had adenocarcinoma histology, 1 patient had mixt histology.

The majority had EGFR mutations (68.6%) followed by ALK (27.6%) and ROS1 (2.6%) rearrangements, respectively. EGFR 19 deletion mutation was detected in 131 (61.8%) patients, 61 (28.8%) patients had EGFR exon 21 L858R mutation and other EGFR mutations were present in 20 patients (9.4%) including EGFR exon 20 insertion in 3 of them (1.4%).

One patient had a BRAF V600E mutation and received dabrafenib plus trametinib as first-line therapy. Another one patient had BRAF V600E with EGFR mutation and this patient received chemotherapy, afatinib, chemotherapy, respectively. Another 2 patients had MET amplification with EGFR mutation. One of these patients received crizotinib as first-line treatment, and the other received chemotherapy, afatinib, and crizotinib, respectively.

Local treatment of BM included surgery in 13.4%, radiation in 77% and no local treatment in 9.6%.

Most patients had also extracranial metastasis (86.6%). The clinicopathological characteristics at baseline are listed in Table 1.

**Table 1:** The clinicopathological characteristics of the patients.

Age (median, range)	58 (20–85) years	
	N	%
< 65 years	219	71.6
≥ 65 years	87	28.4
Gender		
Female	155	50.7
Male	151	49.3
ECOG PS		
0	77	25.5
1	158	52.5
2	51	17
3	15	5
Smoking		
No	175	58
≤ 10 package/year	36	11.9
> 10 package/year	91	30.1
Tumor histology		
Adenocarcinoma	289	94.4
NOS (not other specified)	11	3.6
Squamous cell carcinoma	5	1.6
Mixt (adeno-squamous)	1	0.4
Present driver mutation status		
EGFR	210	68.6
Exon 21	61	28.8
Exon 19 del	131	61.8
Rare	20	9.4
ALK	85	27.6
ROS1	8	2.6
BRAF	2	0.6
Number of BM		
1–3	188	61.6
≥ 4	117	38.4
Extracranial metastases		
Yes	265	86.6
No	41	13.4
Local treatment for BM		
No	28	9.6
Surgery	28	9.6
WBRT	123	42.4
SRS	98	33.8
WBRT plus SRS	2	0.7
Surgery plus SRS	6	2
Surgery plus WBRT	5	1.7
RT after first BM progression	78	
SRS	20	25.6
WBRT	30	38.5
Follow-up	28	35.9
RT after second BM progression	32	
SRS	10	31.3
WBRT	6	18.7
Follow-up	16	50
RT after third BM progression	14	
SRS	6	42.85
WBRT	1	7.15
Follow-up	7	50
1st line treatment		
Erlotinib	115	37.7
Gefitinib	19	6.2
Afatinib	27	8.9
Osimertinib	6	2
CT	51	16.7
Crizotinib	25	8.2
Alectinib	51	16.7
Brigatinib	1	0.3
Ceritinib	4	1.3
Dacomitinib	1	0.3
Dabrafenib plus trametinib	1	0.3
Other	4	1.3
2nd line treatment	118	
Erlotinib	27	22.9
Afatinib	6	5
Osimertinib	41	34.7
CT	20	16.9
Crizotinib	4	3.4
Alectinib	10	8.5
Brigatinib	2	1.7
Lorlatinib	8	2.3
3rd line treatment	38	
Erlotinib	1	2.6
Osimertinib	5	13.1
CT	23	60.5
Crizotinib	1	2.6
Alectinib	2	5.2
Brigatinib	2	5.2
Ceritinib	1	2.6
Lorlatinib	2	5.2
Nivolumab	1	2.6
4th line treatment	9	
Erlotinib	2	22.2
Afatinib	1	11.1
CT	4	44.5
Alectinib	2	22.2

*PS* performance status, *BM* brain metastasis, *RT* radiotherapy, *SRS* stereotactic radiosurgery, *WBRT* whole-brain radiotherapy, *CT* chemotherapy.

The median duration of follow-up was 33.1 months (95% CI, 26.8–39.5). During the follow-up period, an event of disease progression had occurred in 228 patients and death had occurred in 155 patients. The cause of death was reported as brain progression in 45 patients.

**Figure 1:**
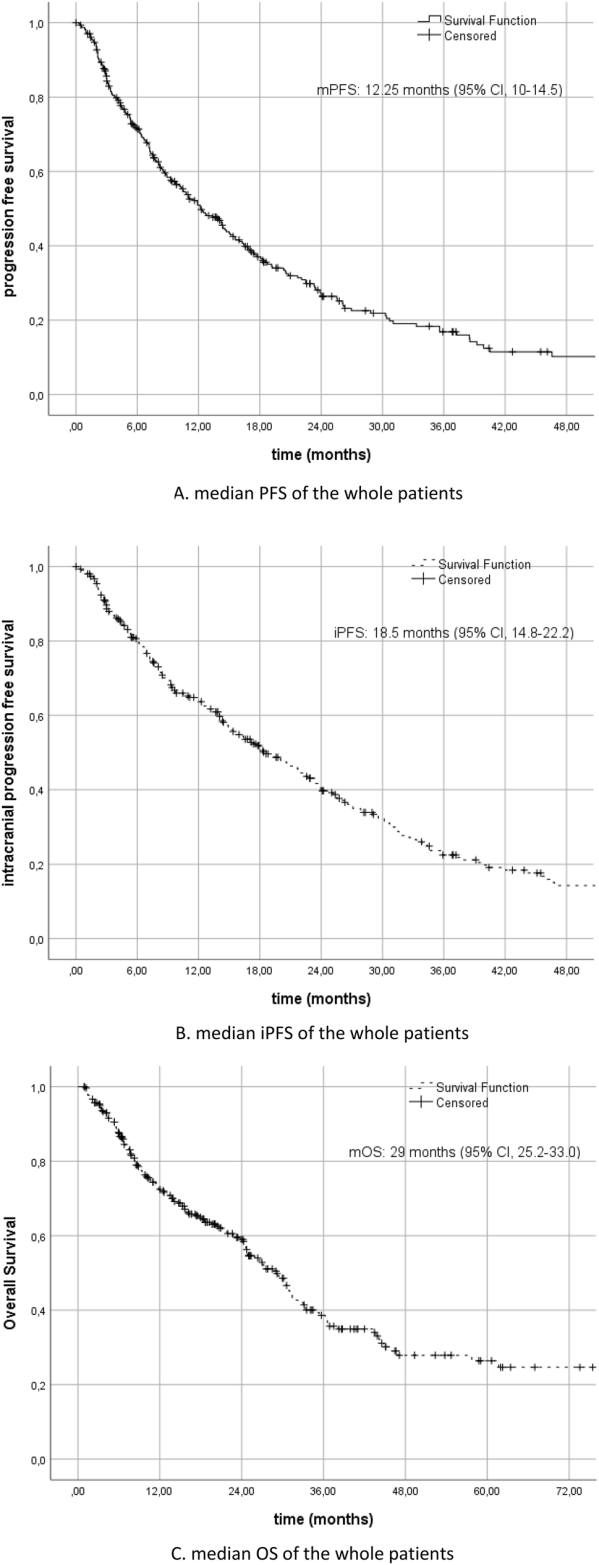
(**A**) Median PFS of the whole patients. (**B**) Median iPFS of the whole patients. (**C**) Median OS of the whole patients.

The median PFS was 12.25 months (95% CI, 10–14.5) (Fig. 1A). While 254 of the patients received TKIs, 51 patients received chemotherapy (CT) as first line treatment. After learning the mutation results of 10 patients who had started on CT, CT was stopped and switched to TKI treatment. 118 patients received 2nd line treatment and 88 of them had disease progression. 43 patients received subsequent therapies and 30 of them had disease progression. CNS disease progression developed in 209 patients. The median iPFS was 18.5 months (95% CI, 14.8–22.2) (Fig. 1B). The median OS was 29 months (95% CI, 25.2–33.0) (Fig. 1C).

**Figure 2:**
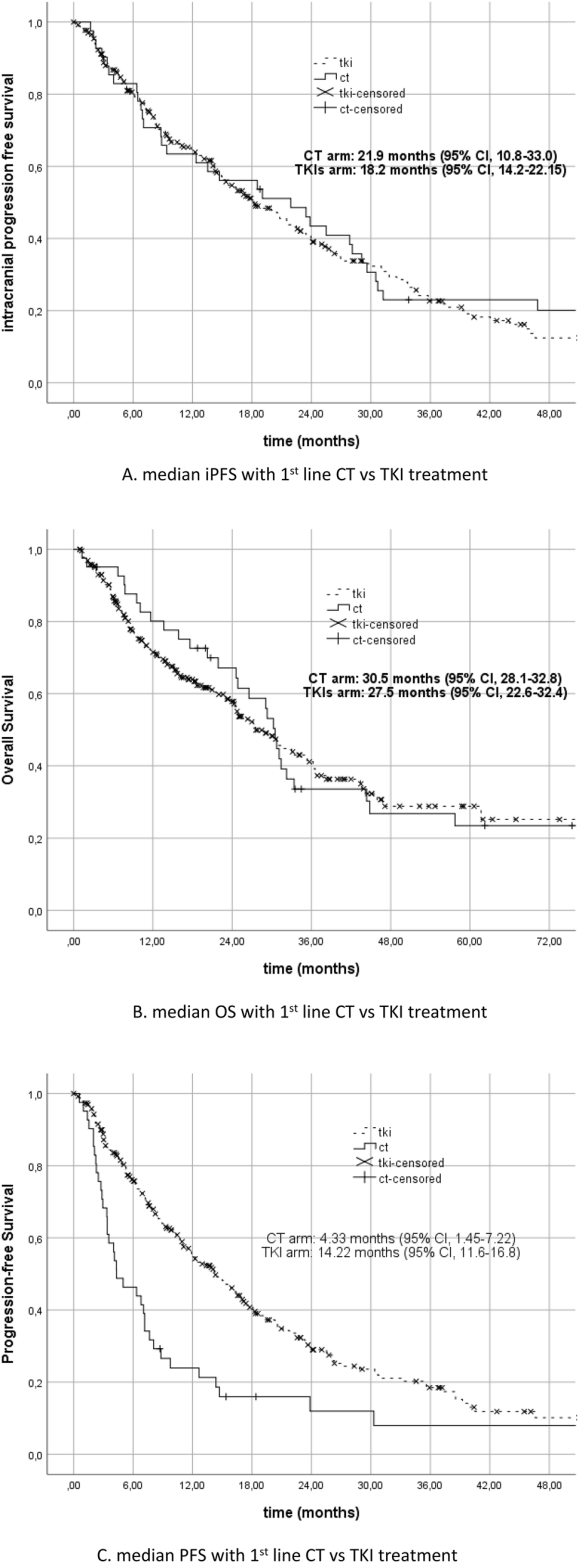
(**A**) median iPFS with 1st line CT vs TKI treatment. (**B**) median OS with 1st line CT vs TKI treatment. (**C**) median PFS with 1st line CT vs TKI treatment.

In this group of patients receiving sequential therapy, there was no difference in terms of iPFS and mOS between patients who received CT as a first-line treatment and those who started with a TKI (*p* = 0.68 and *p* = 0.70, respectively) (Fig. 2A,B). However, there was a significant improvement with the first-line TKI treatment in terms of mPFS (*p* < 0.001) (Fig. 2C).

**Figure 3:**
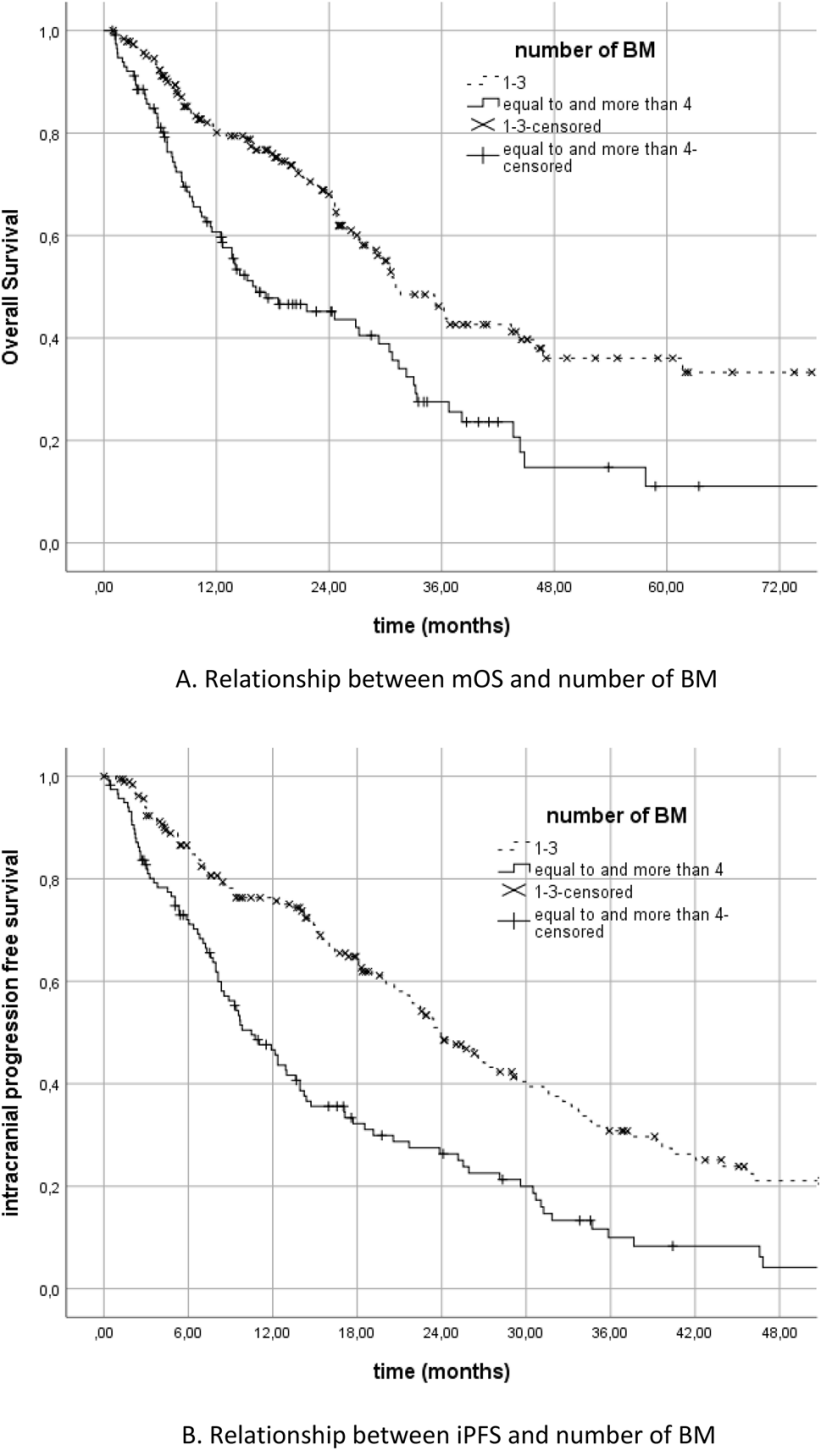
(**A**) Relationship between mOS and number of BM. (**B**) Relationship between iPFS and number of BM.

It was found to be significantly associated with the number of BM and mOS. Patients with 3 or less BM had better survival than patients with 4 or more BM (31.1 (95% CI, 25.2–37.0) vs 16.2 (95% CI, 7.3–25.2) months, *p* < 0.001) (Fig. 3A). And in terms of iPFS, the patients with 3 or less BM had better survival than the patients with 4 or more BM (23.9 (95% CI, 20.4–27.5) vs 10.5 (95% CI, 8–12.9) months, *p* < 0.001) (Fig. 3B).

**Figure 4:**
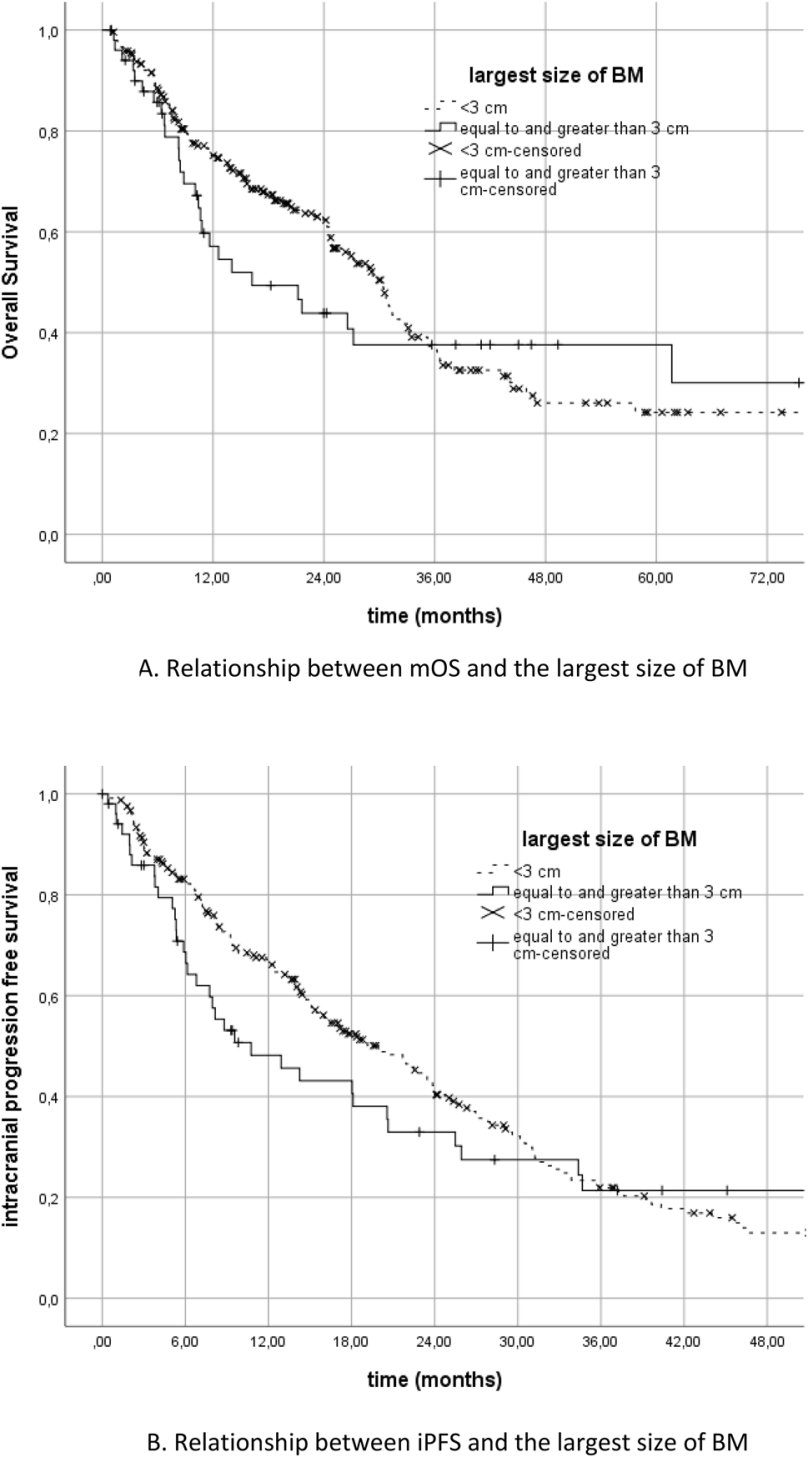
(**A**) Relationship between mOS and the largest size of BM (**B**) Relationship between iPFS and the largest size of BM.

The relationship between the size of BM and survival was found to be non-significant. There was no statistically significant difference in terms of mOS or iPFS when compared between patient groups with the largest BM size less than 3 cm and ≥ 3 cm (*p* = 0.55 and *p* = 0.32, respectively) (Fig. 4A,B).

**Figure 5:**
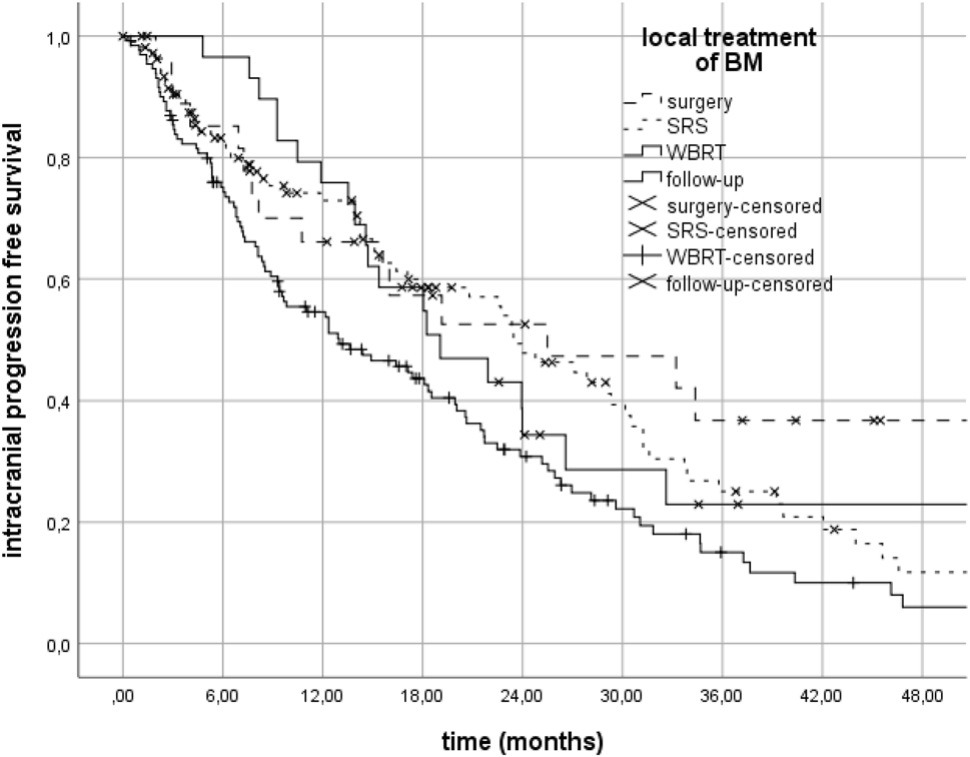
Relationship between iPFS and local treatments of BM.

The effect of local treatment types given for BM on survival was evaluated and the patients treated with surgery and SRS had better survival than the patients receiving WBRT (Fig. 5). While 80 of the patients who received WBRT treatment had 1 to 3 BM, 42 of them had ≥ 4 BM, and iPFS was significantly better for the group of patients with 1–3 BM (Table 2).

**Table 2:** median iPFS according to local treatment modalities of BM.

Local treatment of BM	iPFS (months)	*p*
Surgery	25.5 (2.5–48.4)	S vs WBRT: 0.01
SRS	23.4 (18.1–28.7)	SRS vs WBRT: 0.008
1–3 BM	24.8 (18.8–30.7)	0.08
≥ 4 BM	14.2 (4.8–23.6)	
WBRT	13 (7–19)	
1–3 BM	20.6 (16.3–25)	0.01
≥ 4 BM	9.5 (6–13)	
Follow-up	19 (11.3–26.8)	

*BM* brain metastasis, *S* surgery, *SRS* stereotactic radiosurgery, *WBRT* whole-brain radiotherapy.

Regarding the patients with EGFR mutation, while iPFS (*p* = 0.14) and mOS (*p* = 0.30) were not statistically different between the patient groups carrying exon 19 deletion, exon 21 mutation or other rare EGFR mutations; both iPFS and mOS were numerically shorter in rare EGFR mutation carriers (Table 3).

**Table 3:** median iPFS and mOS according to EGFR mutations.

EGFR mutation	iPFS	mOS
Exon 21	14.9 (13.3–16.5)	24.8 (16.2–33.4)
Exon 19	20.8 (15.8–25.8)	27.2 (22.3–32)
Rare	3 (0.1–9.1)	12.5 (5.5–19.5)

In EGFR mutant population, number of BM was associated with iPFS (22.7 vs 9.7 months, *p* < 0.001) and mOS (29.6 vs 14.0 months, *p* < 0.001). No correlation was shown between size of BM and survival parameters. While no statistical difference was found between the presence of extracranial metastases and mOS, iPFS was better in patients without extracranial metastases than in those having extracranial metastases.

In ALK positive population, number of BM was associated with iPFS (37.2 vs 14.4 months, *p* = 0.002) and mOS (61.7 vs 33.2, *p* = 0.004). No correlation was shown between size of BM and survival parameters. Presence of extracranial metastases was associated with survival outcomes and iPFS and mOS were better in patients without extracranial metastases than in those having extracranial metastases. In Table 4, the results of subgroup analysis of EGFR mutant and ALK positive patients according to BM status were summarized.

**Table 4:** Subgroup analysis of EGFR and ALK mutant patients regarding iPFS and mOS and associated variables.

	iPFS (months)	*p*	mOS (months)	*p*
EGFR mutant patients				
Number of BM				
1–3	22.7 (19.2–26.2)	** < 0.001**	29.6 (26–33.2)	** < 0.001**
≥ 4	9.7 (7.5–11.9)		14 (10.5–17.5)	
Size of BM				
< 3 cm	17.7 (12.9–22.5)	0.35	27.2 (22.8–31.5)	0.78
≥ 3 cm	10.7 (4.3–17.2)		16.2 (3.7–28.7)	
Presence of extracranial metastases				
No	27.1 (13.2–41)	**0.03**	24.9 (11.1–38.7)	0.59
Yes	16.4 (12.6–20.2)		26.5 (22.9–30.1)	
ALK mutant patients				
Number of BM				
1–3	37.2 (23.9–50.6)	**0.002**	61.7 (NE-NE)	**0.004**
≥ 4	14.4 (3.9–24.9)		33.2 (21.8–44.6)	
Size of BM				
< 3 cm	23.4 (16.1–30.8)	0.65	44.3 (31.7–57)	0.58
≥ 3 cm	52.5 (1.8–103.3)		61.7 (0.1–140.1)	
Presence of extracranial metastases				
No	87.16 (NE-NE)	**0.001**	NE	**0.008**
Yes	19.15 (10.7–27.6)		NE	

Cox analysis showed that age, ECOG PS, and presence of extracranial metastases were associated with mOS. Age and presence of extracranial metastases were associated with iPFS, but no correlation was found between ECOG PS and iPFS (Table 5).

**Table 5:** Factors associated with iPFS and mOS (cox analysis).

	HR	95% CI	*p*
mOS associated variables			
Age			
< 65			**0.001**
> 65	1.8	1.3–2.5	
ECOG PS			
0–1			**0.02**
> 2	1.54	1.01–2.21	
Extracranial metastasis			
No			**0.048**
Yes	1.7	1.0–2.87	
iPFS associated variables			
Age			
< 65			**0.005**
≥ 65	1.5	1.1–2.1	
ECOG PS			
0–1			0.32
≥ 2	1.18	0.85–1.63	
Extracranial metastasis			
No			** < 0.001**
Yes	2.4	1.5–3.9	

## Discussion

For NSCLC patients presenting with BM, knowledge of the driver gene mutation status is particularly important because such as EGFR, ALK, and RET has reported to be the risk factors contributing to BM development in advanced NSCLC^17^. It is currently possible to talk about long survival data, especially thanks to the effectiveness of TKIs that crosses the blood–brain barrier and delays both the emergence and progression of BM. In addition, improvements in the field of radiotherapy techniques for BM have resulted in a positive contribution to survival^18^.

In our study, upfront TKIs and starting chemotherapy as the first-line therapy were not different in terms of long-term CNS-related or overall survival. In contrast, superior efficacy of EGFR-TKIs than chemotherapy as the first-line therapy has been shown^19,20^. The potential difference may have been obscured by the factors that the patients in this cohort must have received at least 1 line of TKI therapy, the ability to switch to TKI treatment immediately after progression during chemotherapy and also application of local treatment for BM despite shorter mPFS with chemotherapy.

90% of our patients, all of whom presented with BM at the time of diagnosis, initially received local treatment for BMs and all patients were started on systemic treatment. The reason why iPFS was longer than systemic progression in our study can be interpreted as the effect of local treatments and systemic treatment together on BM control. In case of progression of BM during follow-up, if the isolated brain metastasis progressed, they were followed with a second local treatment without changing the systemic treatment. However, if prominently symptomatic, multiple BM or accompanying systemic progression occurred, systemic treatments were changed and the next treatment step was started. Since reflecting our real-life practice in the general population, the results of our study are valuable.

In the context of combining/sequencing local treatments and TKI treatments for BM in the patients with driver mutant NSCLC, when survival-related factors are evaluated either considering the EGFR mutant and ALK positive patient groups separately or the entire group, having 1–3 BMs and isolated BMs were associated with significantly better iPFS and OS data, in line with the literature^21^. In general, in this the group of patients with measurable BM, the size was not at all associated with survival. As a limitation of our study, the BM volumes of the patients were not recorded. We believe that assessing the relationship between number, size and volume of BMs with prospective studies would be valuable for the patients with driver mutant NSCLC with BMs.

Regarding the efficacy of local treatments on survival parameters, the patients who underwent surgery and SRS had similar iPFS and mOS, while the survival of the patients who received WBRT was inferior when compared to surgery/SRS. Although two third of the patients who were treated WBRT had 1–3 BM, it was observed that the results of WBRT were not better. For the patient group with a low number of BM, WBRT does not seem to provide additional contribution compared to SRS. A recent retrospective Korean study reported that RT was associated with a survival difference among Korean patients with BM, especially among patients with BM from lung cancer and also that SRS was associated with better overall survival, relative to WBRT^22^.

When evaluating the patients with EGFR mutation subtypes in particular, mutation subtypes did not seem to have an independent prognostic impact on survival. Although the survival results of exon 19 deletion mutation carriers were numerically better, there was no statistical difference between exon 19 deletion and exon 21 mutation carriers in terms of iPFS or mOS. On the contrary, the patients with other EGFR mutations (such as exon 18, exon 20 mutation, overexpression, amplification) had numerically inferior survival results, however no statistically significant difference was found again.

According to the results of another retrospective, non-interventional study with a similar design to ours, EGFR mutations other than exon 19 deletion EGFR mutations were associated with earlier intracranial progression and iPFS was significantly better in patients with EGFR exon 19 deletions compared to patients with other EGFR mutations^23^. However, it would be more informative to specify the specific subtypes of EGFR mutations for 43% of patients reported to have non-deletion 19 EGFR mutation. Results of the ongoing prospective studies evaluating more potent monoclonal antibody and TKIs for the patients with other EGFR mutation (especially other than exon 19 deletion and exon 21 mutation and for exon 20 ins mut) will be instructive in regard to CNS metastasis.

For the whole patient group, BM less than 4, absence of extracranial metastases, good performance status at the time of admission (asymptomatic or minimally symptomatic) and age below 65 were observed as predictive markers for better survival data. The fact that number of BM is a prognostic factor rather than size may be related to better local control of BM in these patients.

As we know that almost 30–40% of patients presenting with de novo BM are included and evaluated in pivotal studies; our study is important because all patients included were diagnosed with oncogene-driven NSCLC presented with de novo brain metastases and reflects real-life data despite possible biases regarding retrospective nature. Another limitation of our study is that the evaluation of BM with radio-imaging modality was performed in different tertiary centers and radiologist and not provided by a single center standard. Additionally, it could not provide information about the optimal timing of radiotherapy. Additionally, only clinical information of patients with existing driver mutations was included. Therefore, there is no extensive knowledge on whether patients received NGS, the presence of additional mutations, and its relationship with clinical factors.

However, according to our knowledge, this study represents one of the largest real-world studies evaluating advanced NSCLC patients with de novo BM. In line with the literature, the number of BM less than 4 and the absence of extracranial metastases have been found as the factors associated with better survival outcomes.

ALK-positive patients received crizotinib, alectinib, brigatinib, and ceritinib for the first-line. Patients could receive lorlatinib in the 2nd and 3rd line due to the lack of reimbursement in our country. The results of our study which included first line chemotherapy, crizotinib and sequential ALK inhibitor treatment receiving patient population, could be interpreted as consistent with the literature. In further studies, to what extend local treatments will contribute to survival in conditions where newer generation TKIs are used more intensively may be the subject of research.

As a therapeutic target, epidermal growth factor receptor (EGFR) mutations, anaplastic lymphoma kinase (ALK) rearrangements, c-ros oncogene 1 (ROS1) rearrangements, v-Raf murine sarcoma viral oncogene homolog B1 (BRAF) mutations, Kirsten rat sarcoma viral oncogene homologue (KRAS) mutations, neurotrophic receptor tyrosine kinase (NTRK) 1/2/3 rearrangements, rearranged during transfection (RET) rearrangements, N-methyl-N0-nitroso-guanidine human osteosarcoma transforming gene (MET) exon14 skipping mutations, and activating human epidermal growth factor receptor 2 (HER2) mutations are sought as treatment targets and TKIs are preferred for the advanced stage NSCLC treatment even BM presence^24^. Cytotoxic chemotherapy and early generation TKIs generally failed to achieve therapeutically relevant concentrations in the CNS due to their inability to cross the blood–brain barrier. Several novel and newer-generation TKIs with improved CNS penetrance are currently used in the clinical practice. Erlotinib, gefitinib (1st generation), and afatinib (2nd generation) have intracranial activity in NSCLC patients with EGFR mutations, with objective response rates ranging from 60–80%^25^. However, Osimertinib (3rd generation) was significantly associated with better hazard ratio (HR) for CNS progression-free survival (0.48; 95% CI: 0.26–0.86) compared with gefitinib or erlotinib as the first-line treatment^26^.

Currently, there are five ALK-TKIs for the treatment of ALK-positive NSCLC, namely crizotinib (1st generation), alectinib, ceritinib, brigatinib (2nd generation), and lorlatinib (3rd generation). Ceritinib (45%) and brigatinib (42–67%) demonstrated high intracranial ORR in patients who relapsed after first line treatment with crizotinib^27^. Alectinib demonstrated an 81% of intracranial response among the patients with previously untreated BM^28^. Finally, lorlatinib had 42–48% intracranial response in patients with recurrence after first line crizotinib and reached 82% as a first line treatment^16,29^. According to the results of the CROWN study, the HR for time to intracranial progression for lorlatinib versus crizotinib was 0.10 (95% CI 0.04–0.27) in patients with baseline BM, and 0.02 (95% CI 0.002–0.14) in patients without baseline BM^16^. Studies are encouraged to clarify the sequence in which ALK-TKIs should be used for effective BM control.

Novel therapeutic strategies following osimertinib resistance are also being investigated. Beyond BM progression, osimertinib-based combination strategy can be considered as one of them. In addition, efforts are being made to develop biomarker-focused treatment strategies for patients harboring a definite acquired resistance alteration, such as MET amplification^30^.

In the MARIPOSA trial, the risk of disease progression or death was significantly reduced in patients who received amivantamab (the EGFR-MET bispecific antibody) combined with a brain-penetrant irreversible third-generation EGFR TKI, lazertinib, compared with patients received osimertinib (hazard ratio [HR] 0.70; 95% CI, 0.58–0.85; *p* < 0.001)^31^. FLAURA 2 reported that osimertinib plus platinum-pemetrexed demonstrated improved CNS efficacy compared with osimertinib monotherapy (HR for disease progression or death, 0.47 (95% CI, 0.33–0.66)^32^. Primary results from the MARIPOSA 2 trial announced a median intracranial PFS of 12.5 months for amivantamab plus chemotherapy and 12.8 months for amivantamab plus Lazertinib plus chemotherapy versus 8.3 months for chemotherapy following disease progression with osimertinib treatment (HRs of 0.55 and 0.58; *p* = 0.001 and *p* < 0.001, respectively)^33^. Phase II SAVANNAH study, evaluating the combination of osimertinib with the selective MET TKI, savolitinib (NCT03778229), phase III SAFFRON study, evaluating osimertinib plus savolitinib versus platinum-doublet chemotherapy (NCT05261399) in patients with MET-mediated resistance to osimertinib and, other strategies such as development of antibody–drug conjugates (i.e. patritumab deruxtecan) to overcome resistance following EGFR TKI treatment are in progress^34^. The results of further investigations to unravel the complexity of brain metastatic EGFR-mutated NSCLC and optimal treatment sequence are eagerly awaited.

Last of all, multimodality therapy has come into prominence among the patients with advanced NSCLC patients with de novo BMs who carry a driver mutation and received at least one line of targeted therapy and long-term progression-free and overall survival can be achieved with the advent of targeted agents with high CNS efficiency with more conservative and localized radiotherapy methods. In our study, we discussed and revealed the prognostic factors reflected in the survival of these patients with real-life data.

## Data Availability

The datasets generated during and/or analysed during the current study are available from the corresponding author on reasonable request.
